# Genome-wide genetic diversity yields insights into genomic responses of candidate climate-selected loci in an Andean wetland plant

**DOI:** 10.1038/s41598-020-73976-3

**Published:** 2020-10-08

**Authors:** Angéline Bertin, Mara I. Espinosa, Catalina A. Bustamante, Alejandra J. Troncoso, Nicolas Gouin

**Affiliations:** 1 Departamento de Biología, Facultad de Ciencias, Universidad de La Serena, Raúl Bitrán 1305, La Serena, Chile; 2 Departamento de Ciencias, Facultad de Artes Liberales, Universidad Adolfo Ibáñez, Diagonal Las Torres 2640, Santiago, Chile; 3 Instituto de Investigación Multidisciplinar en Ciencia Y Tecnología, Universidad de La Serena, La Serena, Chile; 4 Centro de Estudios Avanzados en Zonas Áridas (CEAZA), Raúl Bitrán 1305, La Serena, Chile

**Keywords:** Evolutionary ecology, Molecular ecology, Population genetics

## Abstract

Assessing population evolutionary potential has become a central tenet of conservation biology. Since adaptive responses require allelic variation at functional genes, consensus has grown that genetic variation at genes under selection is a better surrogate for adaptive evolutionary potential than neutral genetic diversity. Although consistent with prevailing theory, this argument lacks empirical support and ignores recent theoretical advances questioning the very concept of neutral genetic diversity. In this study, we quantified genome-wide responses of single nucleotide polymorphism loci linked to climatic factors over a strong latitudinal gradient in natural populations of the high Andean wetland plant, *Carex gayana*, and then assessed whether genetic variation of candidate climate-selected loci better predicted their genome-wide responses than genetic variation of non-candidate loci. Contrary to this expectation, genomic responses of climate-linked loci only related significantly to environmental variables and genetic diversity of non-candidate loci. The effects of genome-wide genetic diversity detected in this study may be a result of either the combined influence of small effect variants or neutral and demographic factors altering the adaptive evolutionary potential of *C. gayana* populations. Regardless of the processes involved, our results redeem genome-wide genetic diversity as a potentially useful indicator of population adaptive evolutionary potential.

## Introduction

In the current context of unprecedented biodiversity loss^1,2^, assessing the adaptive potential of species and populations has become a central goal in conservation biology^2,3^. Because genetic variation defines opportunities for adaptive evolution^4–6^, it is universally recognized as a crucial population characteristic indicative of adaptive potential^7–9^. Nevertheless, there is some debate as to whether traditional estimates of genetic variation are suitable for this purpose^10^. Many studies have resorted to neutral markers for their convenience^8^. However, neutral genetic diversity has increasingly been dismissed as a useful proxy for adaptive potential^11–13^, since it is not directly targeted by selection and covaries only weakly with quantitative genetic variation^14^.

Dismissing traditional neutral genetic diversity estimates as useful indicators of adaptive potential both negates the possibility that neutral and demographic processes play a role in populations’ capacity to respond to selective pressures and assumes that neutral genetic diversity is well characterized and unimpacted by adaptive processes. Yet, neither of these assumptions is likely to be universally true. While adaptive variation is necessary for populations to adapt to new environmental conditions, there is no one-to-one relationship between adaptive genetic diversity and responses. Both neutral and demographic factors, such as population size and gene flow, can actually alter this relationship^5,15,16^. For instance, successful adaptations to new selective pressures are more likely in large populations than in small ones; this is because large populations hold more absolute copies of potentially favorable alleles, have a lower risk of losing those alleles by drift, and have greater potential for mutation, including beneficial de novo mutations^5^. Gene flow can also either constrain or favor adaptation by causing fitness declines resulting from the introduction of maladapted individuals^17,18^ or, in contrast, by introducing new beneficial genetic variants^19,20^. Thus, in populations where neutral and demographic processes significantly alter adaptive evolutionary capacity, neutral genetic diversity could provide useful insights into adaptive potential. On the other hand, growing evidence indicates that neutral genetic diversity can be influenced by selection at linked loci^21–23^ and that a significant proportion of genetic variants within genomes, originally thought to be neutral, could in fact be under selection and functional as a result of the combined genome-wide influence of small effect variants^24–26^. Thus, genome-wide genetic diversity could also reflect adaptive processes and, as such, be a useful predictor of population adaptive potential.

We investigated this by analyzing the extent to which genomic responses of climate-linked loci relate to the genetic diversity of candidate climate-selected loci as well as genome-wide genetic diversity of non-candidate selected loci over a strong latitudinal gradient, using the high Andean wetland plant *Carex gayana* as a model. A key challenge in these types of studies is how to quantify adaptive responses. Ideally, these should be assessed directly from genes under selection^27^, but such genes are in most cases unknown^11,28^. While this issue has been partially resolved thanks to large-scale next-generation sequencing, which allows candidate-selected loci^12,29^ with generally strong effects to be identified, tracking changes in allele frequencies driven by environmental factors is often impossible, particularly in natural populations. Here, we used multivariate analyses (i.e. co-inertia analysis) of single nucleotide polymorphism (SNP) loci datasets to quantify divergence between candidate climate-selected loci and genome-wide non-candidate loci datasets. By applying this approach, we demonstrate that genome-wide genetic diversity of non-candidate loci is a more powerful predictor of evolutionary responses of climate-linked loci in *C. gayana* than genetic diversity of the loci used to measure these responses.

## Results

**Figure 1:**
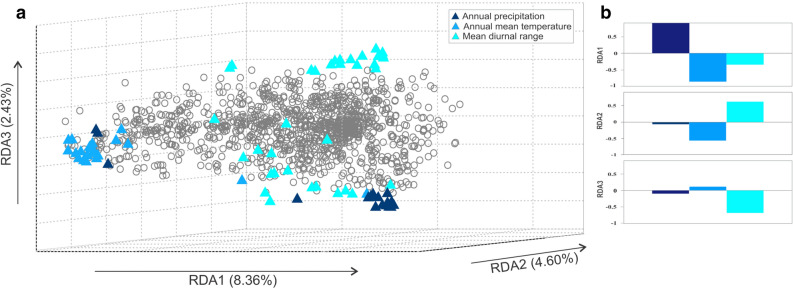
Detection of candidate climate-selected loci by redundancy analysis. (**a**) Projection of the SNP loci on the three canonical axes (RDA1–RDA3) of the redundancy analysis of the genetic data, including annual precipitation, annual mean temperature, and mean diurnal range as explanatory variables. Loci with scores higher than 2.5 SD around the mean (i.e. outlier loci) are plotted in color. The color indicates the climatic variable with which the allele frequency of the candidate climate-selected loci are most correlated. (**b**) Bar plots indicate the weight of the climatic variables on each of the canonical axes.

Using an original database of 1709 SNPs genotyped for 158 specimens by Pfeiffer et al.^30^, we identified candidate climate-selected loci from the WorldClim database based on redundancy analysis (RDA), a genotype-environment association method^31^ linking genotypes at each locus to variables likely to exert selective pressures. The final RDA model used for this purpose (Supplementary Note on variable selection and detection of candidate climate-selected loci) included annual precipitation (BIO12), annual mean temperature (BIO01) and mean diurnal range (BIO02) as predictor variables. The model explained 14% of the total genetic variance (Adjusted *R*^2^ = 0.14, *F*_3,154_ = 9.33, *P* = 0.001) and identified three significant RDA axes (*F*_1,154_ = 4.42–15.20, *P* = 0.001 in all cases) comprising 90 putatively adaptive loci notable for their outlier scores (Fig. 1). Genetic diversity estimates (*He*) of these loci did not correlate with those of the non-candidate loci (Pearson correlation: *r* = 0.30, *P* = 0.25).

**Figure 2:**
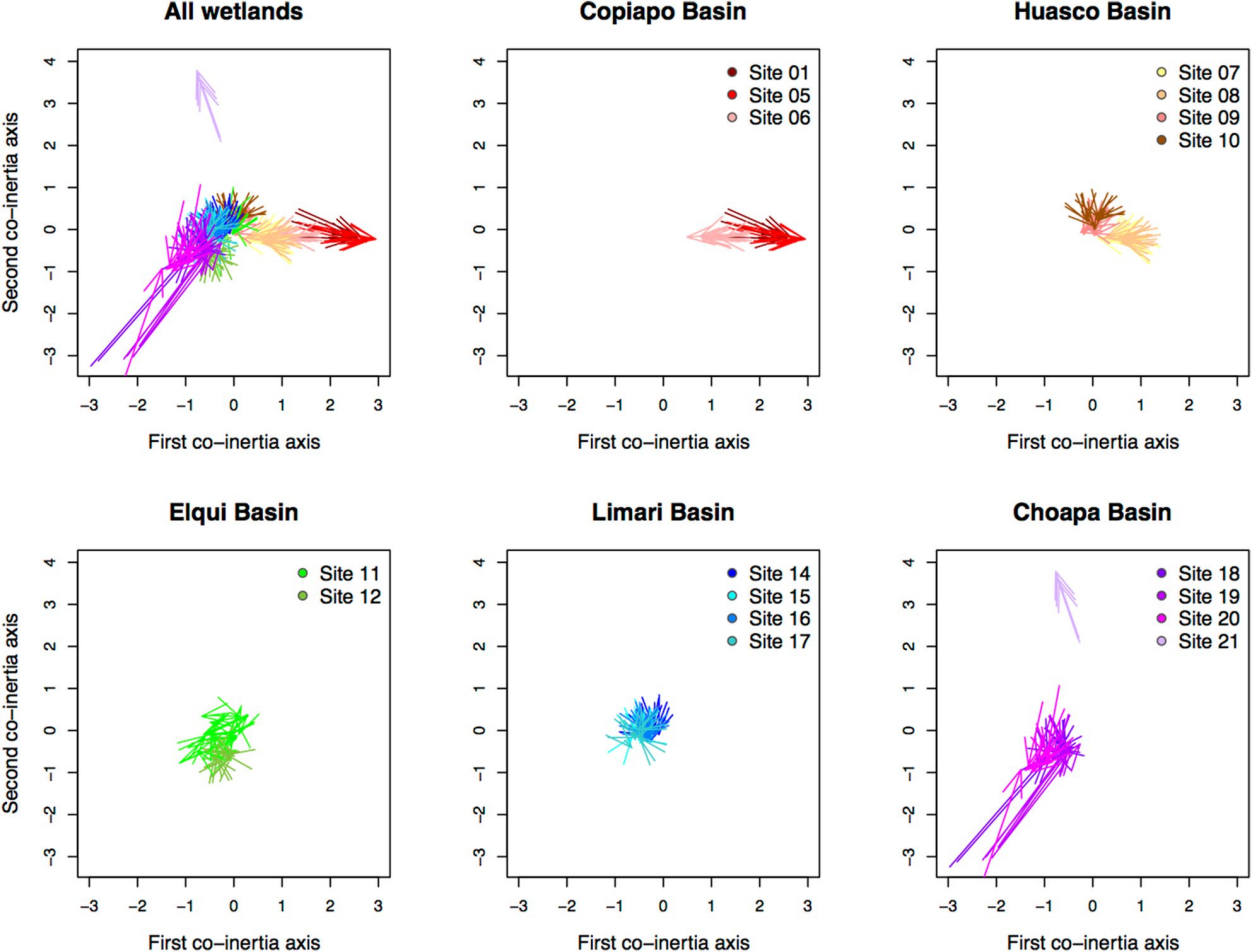
Co-inertia analysis of candidate and non-candidate climate-selected loci of *Carex gayana* considering the entire study area and each river basin separately. Each arrow of the CoIA plots corresponds to a single individual, denoting the position of its genetic data for non-candidate (origins of the arrows) and candidate climate-selected (arrowheads) loci.

By performing a co-inertia analysis^32^ (CoIA), we projected the SNP profiles of the genotyped individuals for the candidate and non-candidate selected loci onto a common multivariate space. For these analyses, we used a dataset composed of 90 loci randomly selected from the full non-candidate loci dataset (comprising 1421 loci, excluding all outlier loci detected using multiple techniques) to ensure a matching number of loci between the non-candidate and candidate selected loci datasets. The CoIA, which captured 80.2% of the total inertia of the non-candidate and candidate selected SNP matrices on its first three axes, characterized spatial genetic structure, showing that individuals from the same wetland, and sites from the same basin, tended to be grouped (Fig. 2). This spatial structure was further confirmed by other analyses of population genetic structure (Supplementary Note on population genetic structure), including AMOVAs, which revealed significant genetic differentiation between basins and between wetlands within basins, both for the non-candidate and candidate selected SNP datasets, with higher levels for the candidate selected loci. Collectively, individuals from the Copiapó and Huasco basins were highly differentiated both from those of the other basins as well as from each other, being most eccentrically positioned relative to the CoIA origin and located in opposing quadrants (Fig. 2). Site 21 in the Choapa basin was atypical, demonstrating marked variation from all other sites, including those from the same basin.

**Figure 3:**
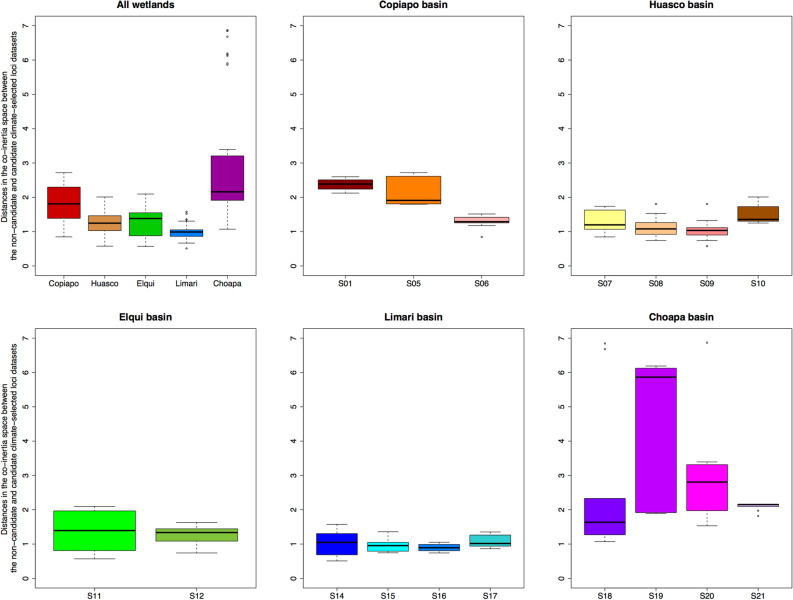
Boxplots of genomic divergence between candidate and non-candidate climate-selected loci datasets per basin and per population in *Carex gayana*, as estimated from Euclidean distances between co-inertia scores of candidate and non-candidate genetic data on the first seven co-inertia axes.

According to the CoIA, the non-candidate and candidate climate-selected SNP datasets were only weakly, although significantly, correlated (*RV* = 0.25, *P* < 0.001). This indicates that a large fraction of the genetic variation of the candidate and non-candidate selected loci did not co-vary. To ensure that this pattern was truly specific to these two datasets, we compared the observed *RV* correlation to a null distribution simulated by a bootstrap procedure from two datasets of 90 loci randomly selected from the full non-candidate loci dataset. None of the 10,000 bootstrap iterations produced an *RV* coefficient as low as that of the candidate and non-candidate selected SNP datasets (Bootstrap RV range: 0.76–0.96, Supplementary Fig. S6). Projecting individuals’ genetic data onto the CoIA space allows divergent patterns between non-candidate and candidate selected loci data to be visualized. Each arrow of the CoIA plots corresponds to a single individual, denoting genetic data positions for non-candidate (origins of the arrows) and candidate climate-selected (arrowheads) loci. Arrow length reflects the amplitude of the divergence between the two datasets, and thus the signature of processes driving divergence between them. Proximity to plot origin and arrow length are indicative of the strength of contribution to the co-variation (total co-inertia) between the two datasets, where individuals located close to plot origins and with short arrows are substantial contributors and *vice versa*^33^. Divergence between the candidate climate-selected and non-candidate loci varied both between basins as well as wetlands (linear model of Euclidean distances between CoIA scores of the candidate and non-candidate loci, basin effect: *F* = 29.71, *df* = 4, *P* < 0.001; nested effect of site within basin: *F* = 3.29, *df* = 12, *P* < 0.001). Candidate climate-selected loci data diverged less strongly from non-candidate ones in the Huasco, Elqui and Limarí basins relative to the northernmost (Copiapó) and southernmost (Choapa) basins (Fig. 3). Extreme divergences were observed in three of the four sites located in the Choapa basin (sites 18, 19 and 20, Figs. 2, 3).

**Figure 4:**
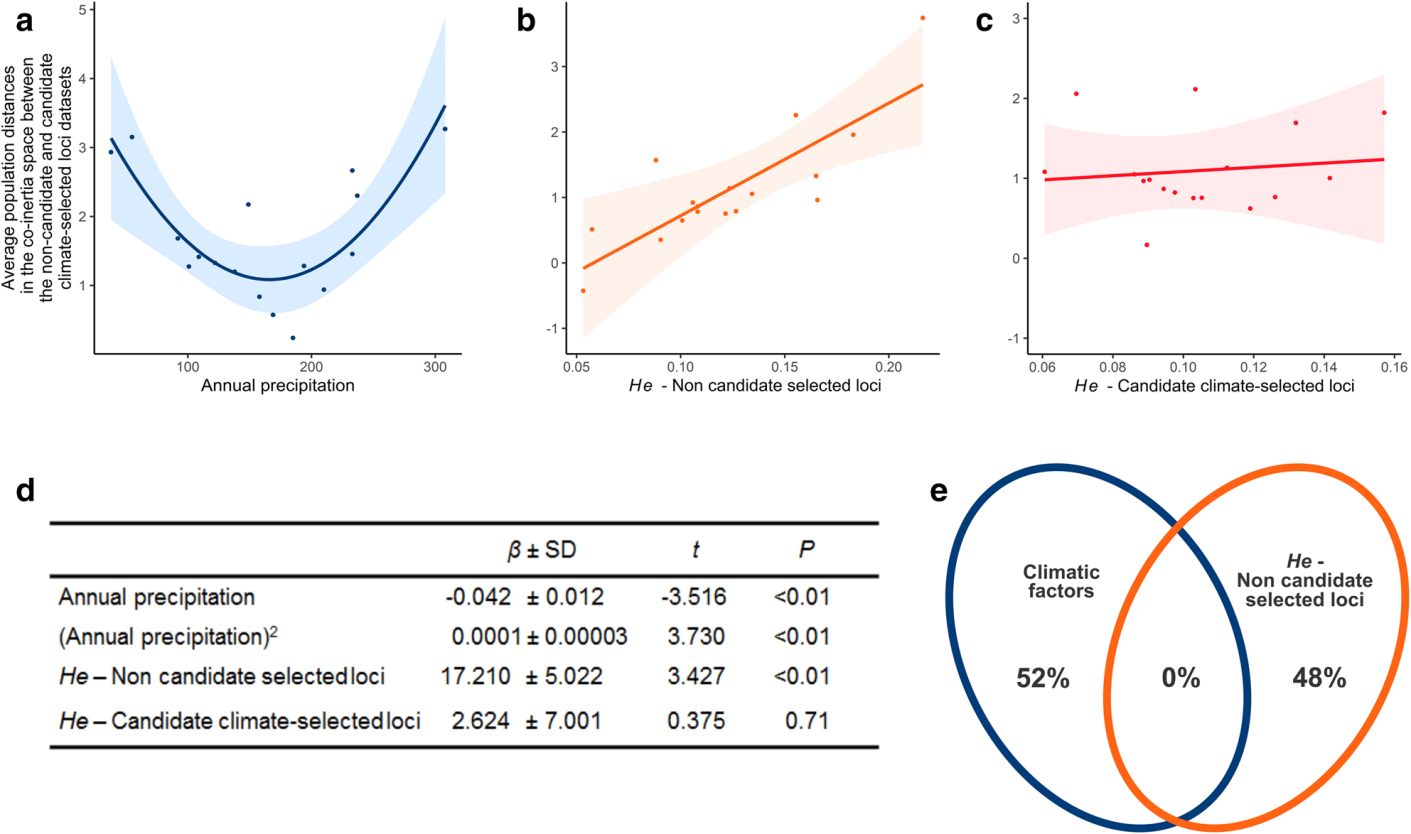
Analysis of the effects of explanatory variables on mean population divergence between candidate and non-candidate climate-selected loci datasets of *Carex gayana*, as estimated from Euclidean distances between the co-inertia scores of each individual on the first seven co-inertia axes for candidate and non-candidate datasets. (**a**–**c**) Conditional plots of the effects of annual precipitation, expected heterozygosity (He) calculated from non-candidate and candidate climate-selected SNP datasets, respectively. (**d**) Results of the regression analysis. (**e**) Proportion of the explained variance in population genetic divergence between candidate and non-candidate selected loci accounted for by significant climatic factors (annual precipitation) and genome-wide genetic diversity of non-candidate selected loci.

Finally, we searched for factors explaining why the candidate and non-candidate selected loci datasets diverged more strongly in some populations than in others. To do that, we first assessed divergence between the two genetic datasets at the population level by averaging divergence estimates of all individuals from the same wetland. Using a linear model, we analyzed the effects of variables that could favor or constrain population divergence between the candidate and non-candidate selected loci datasets. The set of explanatory variables included climatic variables likely to exert selective pressures (i.e. mean annual temperature and precipitation) and genetic diversity of the candidate and non-candidate selected loci. The regression model revealed a significant effect of mean annual precipitation and of genetic diversity of the non-candidate loci. In contrast, no such effects were found with respect to genetic diversity of the candidate climate-selected loci (Fig. 4c–e). Altogether, precipitation data and genetic diversity of the non-candidate loci accounted for 57% of average population divergence between profiles of the candidate and non-candidate selected loci (Adjusted *R*^2^ = 0.57, *F*_4,12_ = 6.28, *P* < 0.01). Effects were entirely independent, with mean annual precipitation explaining slightly more variation in population divergence than non-candidate loci genetic diversity (Fig. 4e). The genomic divergence of the candidate climate-selected profiles gradually increased with genetic diversity of the non-candidate loci (Fig. 4b,d), producing a north to south gradient. Precipitation, in contrast, displayed a quadratic effect, resulting in divergence of relatively similar amplitude between the candidate and non-candidate loci datasets in the northernmost and southernmost sites, characterized by lowest and highest precipitation volumes, respectively (Fig. 4a,d, Supplementary Table S1).

## Discussion

Our results demonstrate that genome-wide genetic diversity was more informative in predicting *C. gayana* genomic responses of climate-linked loci than genetic variation at the loci used to measure this response. This suggests that genome-wide genetic variation may be an important indicator of population adaptive potential, which is also supported by a recent laboratory study demonstrating that it favored evolutionary responses of fitness-related traits in the vinegar fly^22^.

The effects of genome-wide genetic diversity detected in this study may have resulted from the persistence of loci relevant to climate adaptation in the non-candidate loci datasets. Recent theoretical and empirical advances clearly question the definition of neutral genetic diversity, and point towards a larger role of natural selection in genome-wide genetic diversity levels^26^. Indeed, a growing body of evidence indicates that natural selection can impact genetic diversity through its effects on linked neutral diversity^21–23,34^. In addition, it has recently been suggested that genetic variants with small individual effects along the genome, originally considered to be selectively neutral, may in fact be functional as a result of their combined effects^24–26^. Traits important in plant responses to climate change are often controlled by genetic regulatory networks involving complex signaling pathways and the effects of several genes ^35,36^, which suggests that many genetic variants with small effects on adaptive responses to climatic variables could be spread along the genome of *C. gayana*. Since genetic-environmental methods only detect loci with major effects on adaptation^37^, there is a high likelihood that a large number of variants with small individual effects on climate-selected traits were included in the non-candidate climate-loci dataset, and could have contributed to genomic responses of climate-linked loci. Nevertheless, this does not explain why these responses were better predicted by genome-wide genetic diversity than genetic variation at the loci used to measure them, given that an equal number of loci between the two datasets was used.

Constraints imposed by neutral processes on the evolutionary adaptive potential of *C. gayana* could have limited genomic responses of climate-linked loci and could therefore also have contributed to the apparent association of the latter with genome-wide genetic diversity. Since they fuel all genetic variation (be it neutral or adaptive) and can cause its loss^4^, neutral and demographic processes determine the availability of and population capacity to produce new genetic material for selection to act upon. In addition, they can also limit evolutionary adaptive responses even in the presence of substantial adaptive genetic variation^38^, which may occur in the event of demographic constraints^38^ linked, for instance, to small population size, or where gene flow is extensive and outweighs selection effects^19,39,40^. In discrete ecosystems such as high Andean wetlands, neutral processes can strongly impact species and genetic diversity due to greater potential for drift, demographic stochasticity and limited opportunity for dispersal^41^. The importance of neutral processes on genome-wide diversity of *C. gayana* is supported by previous evidence demonstrating strong effects of wetland isolation on this genetic component^42^. Here, we found that high and low precipitation levels were driving genomic responses of climate-linked loci of relatively similar amplitude in the north and south of the study region. Yet, genomic responses of candidate climate-selected loci were more restricted in northernmost wetlands where *C. gayana* populations displayed lower levels of genome-wide genetic diversity. This suggests that demographic constraints could be limiting adaptive potential of this species in the northern sites, which tend to be both smaller and more isolated than the southernmost wetlands^42^.

The poor performance of genetic diversity at candidate climate-selected loci in predicting genomic responses may be attributable to several factors. Firstly, genetic diversity estimates from contemporary samples may inadequately represent levels of genetic variation available at the climate-selected loci prior to selection. Alternatively, this result may indicate that adaptive responses of *C. gayana* populations at climate-selected loci are not solely conditioned by the amount of adaptive genetic variation at loci used to measure this response. As mentioned earlier, there is a high probability that a large number of variants contributing to genomic responses of climate-linked loci (i.e. small effect variants on climate-selected traits) were absent from the candidate climate-loci dataset. In addition, adaptive genetic diversity may not be very informative when de novo mutations make up a substantial part of the evolutionary response, as may be the case in later phases of the adaptive process^16^. Finally, even when adaptation from standing genetic variation dominates, high genetic variation at adaptive loci does not guarantee that variation does exist in the direction of selection and thus may not necessarily facilitate evolutionary adaptive responses. In sum, adaptive genetic diversity may also fail to capture the adaptive potential of populations. Of course, our results do not rule out the utility of adaptive genetic diversity to predict the ability for populations to adapt in situ, nor did they demonstrate the superiority of genome-wide genetic diversity to do so. However, they indicate that this diversity component could be relevant and should not to be discarded. Far more research is needed to understand the circumstances under which genome-wide genetic diversity can be expected to be a reliable predictor of a populations’ adaptive potential.

## Methods

### Sample and SNP data

We used the same SNP dataset used by Pfeiffer et al.^30^, produced by genotyping-by-sequencing^43^ and consisting of 1709 SNPs filtered and genotyped for 158 specimens of the plant *C. gayana* (Cyperaceae) distributed among 17 high Andean wetlands in Chile´s Norte Chico region (latitudinal range: 26S–32S; altitudinal range: 2852–4307 m.a.s.l.). This region is characterized by a large precipitation gradient increasing with latitude, with climatic conditions varying from Mediterranean to hyperarid. Sample size per wetland ranged from 4 to 10 *C. gayana* individuals. Detailed information regarding the study area, study sites, species biology and sampling, DNA extraction and SNP data production can be found elsewhere^30,42,44^. A map of the study area is provided in Supplementary Fig. S5.

### Detection of SNP associated with climatic variables and SNP dataset generation

Statistical analyses for detection of candidate climate-selected loci were performed in the R environment (https://cran.r-project.org). We performed redundancy analyses (RDA) to detect putatively selected loci based on the correlation between individual genotypes and climatic variables potentially acting as selective factors. Prior to this analysis, we used a clustering of variables around latent variables (CLV)^45^ to reduce the initial set of 21 predictor variables, composed of longitude, latitude and 19 standard WorldClim bioclimatic variables (BIO1-19) at 30 s spatial resolution, downloaded from WorldClim version 2^46^ (available at https://worldclim.org/version2). We followed the recommendations of Vigneau et al.^47^ in determining the number of clusters, then selected the variable most representative of each (Supplementary Fig. S1). Details of the environmental and spatial data for each wetland are reported in the Supplementary Note on variable selection and detection of candidate climate-selected loci. RDA was then carried out as described in Forester et al.^31^ using the package vegan^48^. We calculated the variance inflation factors (VIF) to check for multicollinearity problems considering a maximum value of 10. Significance of the RDA model and each individual RDA axis was tested using ANOVA-like permutation tests with 9999 randomizations. We identified outlier loci on each significant axis with a cut-off of 2.5 SD around the mean. Based on these results, we separated the SNPs into candidate climate-selected and non-candidate loci datasets, composed of outlier loci associated with climate variables (i.e. 90) and loci not identified as outliers according to multiple detection methods (i.e. 1421), respectively. In order to remove as many loci with potentially large effects as possible, the non-candidate loci dataset was constructed by eliminating the 229 outliers detected by Pfeiffer et al.^30^, 17 monomorphic loci, and 42 of the 90 climate-selected loci that were unique to the present study, from the 1709 original loci. However, to ensure adequate comparison of variation patterns of candidate climate-selected and non-candidate loci using a matching number of loci, we created a non-candidate dataset by randomly selecting 90 loci out of the 1421 non-candidate loci. This dataset was used in all subsequent analyses. Note that all analyses were also performed using the full non-candidate loci dataset. Patterns detected with the 90 and 1421 non-candidate selected loci were always consistent (Supplementary Fig. S7).

### Genetic structure of the candidate climate-selected and non-candidate loci

Genetic differentiation between wetlands was assessed through various traditional approaches (Supplementary Note on population genetic structure). In addition, we investigated the genetic structure common to both the non-candidate and candidate climate-selected SNP data by carrying out co-inertia analyses (CoIA)^32^. CoIA is a symmetric canonical analysis^33^ that ordinates matching objects (e.g. individuals) from two data matrices, in this instance the non-candidate and candidate climate-selected loci datasets, along successive canonical axes. The CoIA axes are calculated such that congruence between the two tables is maximized, approximated by their squared covariance (i.e. inertia). CoIA first requires separate ordinations of the two matrices, which we performed by subjecting the non-candidate and candidate climate-selected datasets to principal component analyses. The number of principal components that we retained accounted for 90% of the total variation of each dataset. To test the significance of the correlation between the genetic variation of the non-candidate and candidate climate-selected loci datasets, we calculated the RV coefficient, a multivariate generalization of the Pearson correlation coefficient^33^, and tested its significance based on 9999 permutations. Both the principal component and CoIA analyses were performed using the ade4 package^49^. The projection of the genetic profiles of the individuals onto the co-inertia space allows common genetic patterns between the non-candidate and the candidate climate-selected data to be visualized. The distance between the non-candidate and candidate climate-selected dataset in the CoIA space reflects the effects of processes causing the candidate and non-candidate selected loci datasets to diverge. Thus, to quantify the genome-wide signature of these processes, we computed the Euclidean distances between their CoIA scores on the first seven canonical axes, which together represented 94.7% of the co-inertia. We analyzed whether the divergence levels varied geographically by applying a linear model to test for the effects of basins and sites nested within basins.

### Factors linked to divergence between non-candidate and candidate climate-selected loci datasets

We searched for factors linked to population divergence between the non-candidate and candidate climate-selected loci datasets using linear models. To assess divergence between the two datasets at the population level, we averaged divergence estimates of all individuals belonging to the same wetland. The set of explanatory variables included climatic variables potentially acting as selective factors and genetic diversity of candidate and non-candidate selected loci, estimated using gstudio^50^ as the expected heterozygosity (*He*) calculated for each SNP dataset (non-candidate and candidate selected loci, Supplementary Table S5) over all loci with a minimum of four genotyped individuals per population^30^. We used the leaps package^51^ to identify the optimal subset of bioclimatic variables for use in detecting candidate climate-selected loci. We considered that these variables can have both linear as well as quadratic effects. The best model was selected based on a performance analysis returned by leaps using the AICcmodavg package^52^. To estimate the contribution of each of the predictors found to be significant, we performed a variation partition using vegan’s varpart function^48^. Before running these analyses, we ensured that there was no effect of the number of genotyped individuals, as this may act as a possible confounding factor.

## Supplementary information

Supplementary information.

## Data Availability

Raw SNP data files can be found as indicated in Pfeiffer et al.^30^. All other data used in this study are provided in the Supplementary Information file.
