# Supplementary material for: Genome-wide genetic diversity yields insights into genomic responses of candidate climate-selected loci in an Andean wetland plant
Source: Sci Rep. 2020 Oct 8;10:16851. doi: 10.1038/s41598-020-73976-3 (PMC7546723; doi:10.1038/s41598-020-73976-3)
Supplement: Supplementary file 1 — Supplementary information. [file 41598_2020_73976_MOESM1_ESM.pdf]

Supplementary Information

Genome-wide genetic diversity yields insights into genomic responses of candidate climate-selected loci in an Andean wetland plant

Angéline Bertin, Mara I. Espinosa, Catalina A. Bustamante,

Alejandra J. Troncoso, Nicolas Gouin

Content:

SI Note on variable selection and detection of candidate climate-selected loci including Tables S1-S3, Figures S1-S2
SI Note on population genetic structure of the candidate and non-candidate selected loci data including Table S4, Figures S3-S4
Figure S5
Table S5
Figure S6
Figure S7

Variable selection and detection of candidate climate-selected loci

The initial set of environmental variables considered for the outlier detection included latitude, longitude and the 19 standard bioclimatic variables BIO1-19 WorldClim version 2[1] (Supplementary Table S1). Before running the redundancy analysis (RDA) to detect putatively selected loci linked to environmental variables, we reduced the set of environmental variables using the clustering of variables around latent variables (CLV) approach[2]. Following the recommendations of Vigneau *et al.* [3] for determination of the number of clusters, four variables considered to best represent each of the clusters were selected: annual precipitation (BIO12), annual mean temperature (BIO01), mean diurnal range (BIO02) and precipitation of driest quarter (BIO17) (Supplementary Fig. S1). Multicollinearity was evident in the final RDA model integrating these variables, however, indicated by the fact that one of the VIF values was greater than 10. The variable precipitation of driest quarter, which was highly correlated with annual mean temperature (r = -0.68, $P < 0.001$), was therefore eliminated from the analysis. The resulting RDA model presented acceptable VIF values (Supplementary Table S2). The model was highly significant ($P = 0.001$) and explained 14% of the total genetic variance. All three RDA axes were also significant (Supplementary Table S3). In total, 90 loci had outlier RDA scores on these axes, of which 22 correlated most strongly with annual mean temperature, 29 with annual precipitation and 39 with mean diurnal range (Supplementary Fig. S2).

Supplementary Table S1. Spatial and bioclimatic variables considered in the study.

Site	bio01 - Annual Mean Temperature	bio02 - Mean Diurnal Range	bio03 - Isothermality (* 100)	bio04 - Temperature Seasonality (standard deviation *100	bio05 - Max Temperature of Warmest Month	bio06 - Min Temperature of Coldest Month	bio07 - Temperature Annual Range	bio08 - Mean Temperature of Wettest Quarter	bio09 - Mean Temperature of Driest Quarter	bio10 - Mean Temperature of Warmest Quarter	bio11 - Mean Temperature of Coldest Quarter	bio12 - Annual Precipitation	bio13 - Precipitation of Wettest Month	bio14 - Precipitation of Driest Month	bio15 - Precipitation Seasonality (Coefficient of Variation)	bio16 - Precipitation of Wettest Quarter	bio17- Precipitation of Driest Quarter	bio18 - Precipitation of Warmest Quarter	bio19 - Precipitation of Coldest Quarter	Y Coord	X Coord
**1**	8.76	10.76	64.8	224.4	16.82	0.23	16.6	11.24	8.64	11.44	5.83	38	8	1	47.8	17	4	15	10	7006850	465703
**5**	7.49	10.22	59.7	255.6	15.99	-1.12	17.11	10.39	7.22	10.68	4.33	55	11	1	50.3	24	7	21	15	6926610	451469
**6**	4.22	11.13	62.2	251.8	13.18	-4.72	17.9	1.73	3.82	7.38	1.12	92	16	2	50.5	35	11	27	35	6899130	445571
**7**	2.03	12.82	64.6	262.8	11.89	-7.94	19.83	-1.14	3.04	5.33	-1.14	101	18	2	52.1	44	12	23	44	6834860	405773
**8**	1.59	12.72	63.9	268.3	11.43	-8.47	19.9	-0.95	2.66	4.97	-1.64	109	18	3	46.2	44	14	26	44	6830540	411431
**9**	1.75	11.8	59.8	295.2	11.78	-7.96	19.75	-1.11	2.89	5.47	-1.8	122	20	4	47.1	52	17	23	52	6737990	395071
**10**	0.37	14.36	63.2	310	11.89	-10.84	22.74	-2.61	1.57	4.24	-3.4	149	26	4	56.5	69	19	26	69	6723560	403227
**11**	-1.01	14.2	62.8	313.2	10.23	-12.4	22.63	-3.99	0.19	2.87	-4.82	169	28	6	50.1	74	23	30	73	6698510	409920
**12**	3.22	9.04	50.8	323.4	12.22	-5.58	17.8	-0.02	4.62	7.3	-0.69	138	25	4	56	64	17	22	64	6682290	399754
**14**	1.76	10.2	53.1	339.1	11.4	-7.8	19.2	-2.36	5.01	5.95	-2.36	158	30	3	69.8	82	14	16	82	6617890	375395
**15**	1.05	10.46	52.4	360	11.34	-8.63	19.97	-3.32	4.46	5.46	-3.32	185	37	3	75.8	100	14	16	100	6589920	369895
**16**	0.55	11.23	53	376.8	11.18	-10.01	21.19	-2.97	4.09	5.13	-4.05	194	36	4	71.6	100	16	17	99	6569350	373680
**17**	0.83	10.4	51	379.8	11.18	-9.2	20.37	-3.75	5.45	5.45	-3.75	210	45	2	83.1	116	12	12	116	6539470	351580
**18**	-0.73	10.04	50.3	377.2	9.03	-10.96	19.99	-4.23	3.84	3.84	-5.31	233	45	3	78.6	123	14	14	123	6519340	350072
**19**	-0.25	10.81	51.5	387.3	10.01	-11	21.01	-4.98	4.42	4.42	-4.98	233	45	3	80.4	125	13	13	125	6509580	351294
**20**	-0.36	7.25	42.6	370.5	7.96	-9.09	17.04	-4.88	4.13	4.13	-4.88	237	44	3	78.1	124	14	14	124	6502370	347386
**21**	-1.19	10.42	48.1	427.2	9.38	-12.3	21.67	-5.16	3.91	3.91	-6.42	308	60	4	84.4	169	14	14	163	6432710	371258

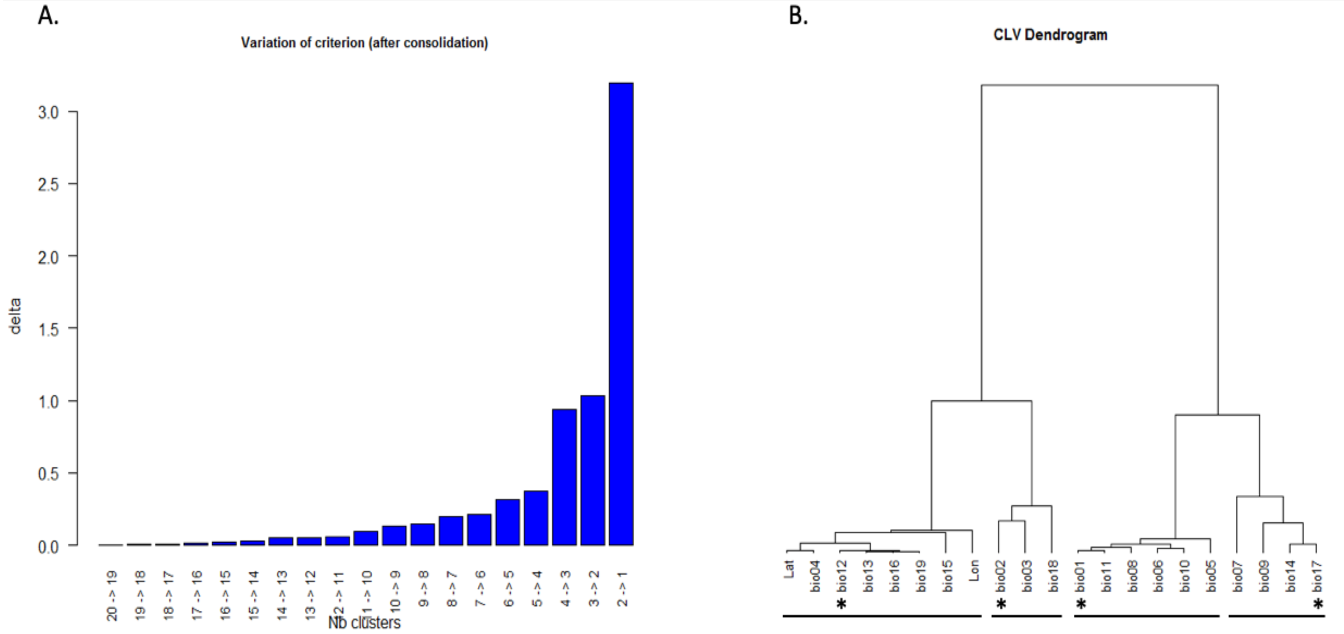

Supplementary Fig. S1. Clustering of variables around latent variables for the 21 environmental and spatial variables considered in the search of outlier loci in *Carex gayana* from 17 high-altitude wetlands across Chile's Norte Chico. **A**. Graph of the variation of the clustering criterion after consolidation. **B**. Dendrogram of the hierarchical clustering of variables around latent variables; horizontal black lines depict the four retained clusters. In each group, the variable that most strongly correlated with that group's latent variable was retained, indicated by an asterisk.

Supplementary Table S2. Variance inflation factors (VIF) of the redundancy analysis of genetic data, including annual precipitation, annual mean temperature, and mean diurnal range as explanatory variables.

**Reduced variables**	**VIF**
Annual mean temperature	6.3
Mean diurnal range	2.2
Annual precipitation	6.9

Supplementary Table S3. Genetic variance explained (adjusted R^2) by the three canonical axes of the redundancy analysis of the genetic data, including annual precipitation, annual mean temperature, and mean diurnal range as explanatory variables.

	% R_{adj}^2	$\boldsymbol{F}$	**d.f.$_{num}$, d.f.$_{den}$**	$\boldsymbol{P}$
RDA1	7.6	15.2	1, 154	0.001
RDA2	4.2	8.4	1, 154	0.001
RDA3	2.2	4.4	1, 154	0.002

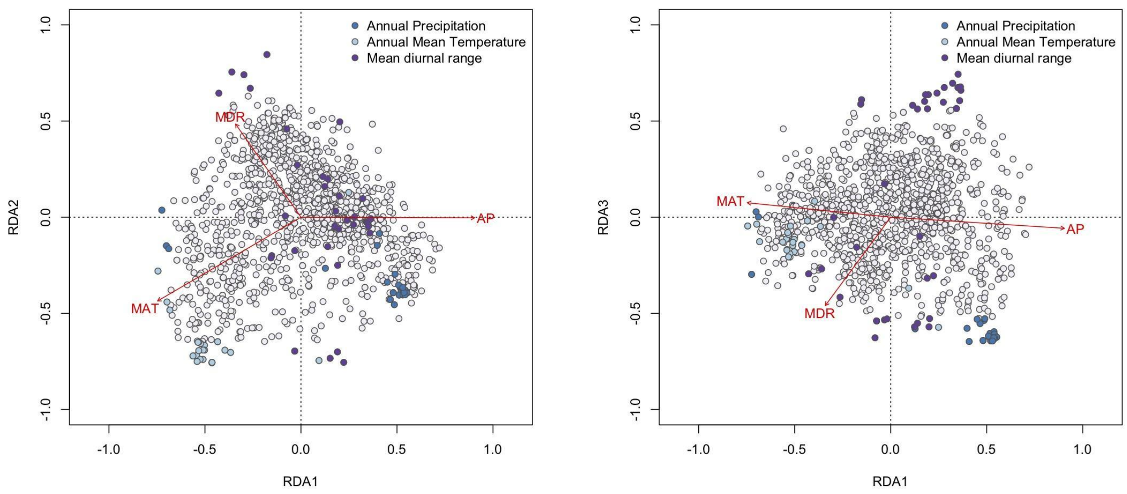

A. B.

Supplementary Fig. S2. Projection of the SNP loci on the three canonical axes (A: RDA1-RDA2 and B: RDA1-RDA3) of the redundancy analysis of the genetic data, including annual precipitation, mean annual temperature, and mean diurnal range as explanatory variables. Loci with scores higher than 2.5 SD around the mean (i.e. outlier loci) are plotted in color. The color indicates the climatic variable with which outlier allelic frequency is most correlated. Red arrows indicate the weight of the climatic variables, MDR = mean diurnal range, MAT = mean annual temperature, AP = annual precipitation.

Population genetic structure analysis of the candidate and non-candidate climate-selected loci

Methods

Genetic differentiation between sample sites was assessed for candidate and non-candidate selected loci datasets using various approaches. We calculated pairwise F_{ST} estimates with Arlequin V3.5.2.2[4] using 9 999 permutations to assess statistical significance, and Principal Coordinate Analysis (PCoA) implemented in GenAlEx 6.5[5] to explore divergence between populations. We additionally performed Discriminant Analysis of Principal Components (DAPC) [6] with the Adegenet v2.0.0 package[7] in R, using each wetland as a predefined group. We used a cross-validation procedure[3] to determine the number of principal components (PCs) retained for the discriminant analysis. Analysis of Molecular Variance (AMOVA)[4] was also performed to assess and test genetic variance partitioning between and within river basins.

Results

All the results show that *Carex gayana* populations are highly differentiated along the study area for both candidate and non-candidate climate selected SNP loci (Supplementary Table S4), with average F_{ST} estimates across all populations of 0.576 and 0.312, respectively, which is similar in magnitude to our previous results based on AFLP markers[8]. Both the PCoA and DAPC analyses performed on the non-candidate selected SNP datasets revealed that all populations are globally well-differentiated (Supplementary Figures S3A, S4A and S4B). The DAPC analysis showed high discriminant power for all sites (Supplementary Figure S4B), with slightly lower discrimination levels between sites S07 and S08, which

appeared to be the least genetically differentiated wetlands in our study (F_{ST} non-candidate selected SNPs = 0.065, $P = 0.019$; F_{ST} candidate climate-selected SNPs = 0, $P > 0.05$). Both analyses also revealed clear genetic differentiation between sites located in the Copiapo and northern Huasco basins (i.e. S01-S08) in the north of the study area relative to the remaining sites (Supplementary Figures S3A and S4A). Further population genetic structure was identified by the AMOVA, with 16.2 to 17.5% of genetic variation significantly distributed between populations from the different river basins (Supplementary Table S4). Also, these analyses clearly show that higher levels of genetic differentiation are found in the candidate climate-selected SNP loci as opposed to the non-candidate selected ones, both over all sites as well as between sites within the same river basins (Supplementary Table S4). Interestingly, the most peripheral populations along our study area appeared to be most differentiated at the candidate climate-selected loci (i.e., the southernmost Choapa site S21 and the northernmost Copiapo sites S01 and S05; Supplementary Figure S3B). All these results corroborate trends detected by co-inertia analysis reported in the main body of the manuscript.

Supplementary Table S4. Analysis of molecular variance (AMOVA) based on the main river basins sampled along the study area for candidate and non-candidate climate-selected loci datasets in *Carex gayana*, each composed of 90 SNPs.

Source of variation	d.f.	Sum of squares	Variance Components	Percentage of variation (%)	*Φ* statistics
**Non-candidate selected loci dataset**					
Among river basins	4	133.4	0.69	16.2	Φ_{CT} = 0.162***
Among sites within river basins	12	140.8	0.99	23.5	Φ_{SC} = 0.281***
Within sites	141	360.4	2.56	60.3	Φ_{ST} = 0.397***
**Candidate climate-selected loci dataset**					
Among river basins	4	124.7	0.56	17.5	Φ_{CT} = 0.175***
Among sites within river basins	12	159.9	1.30	40.1	Φ_{SC} = 0.486***
Within sites	141	193.4	1.37	42.4	Φ_{ST} = 0.576***

***P-value < 0.001

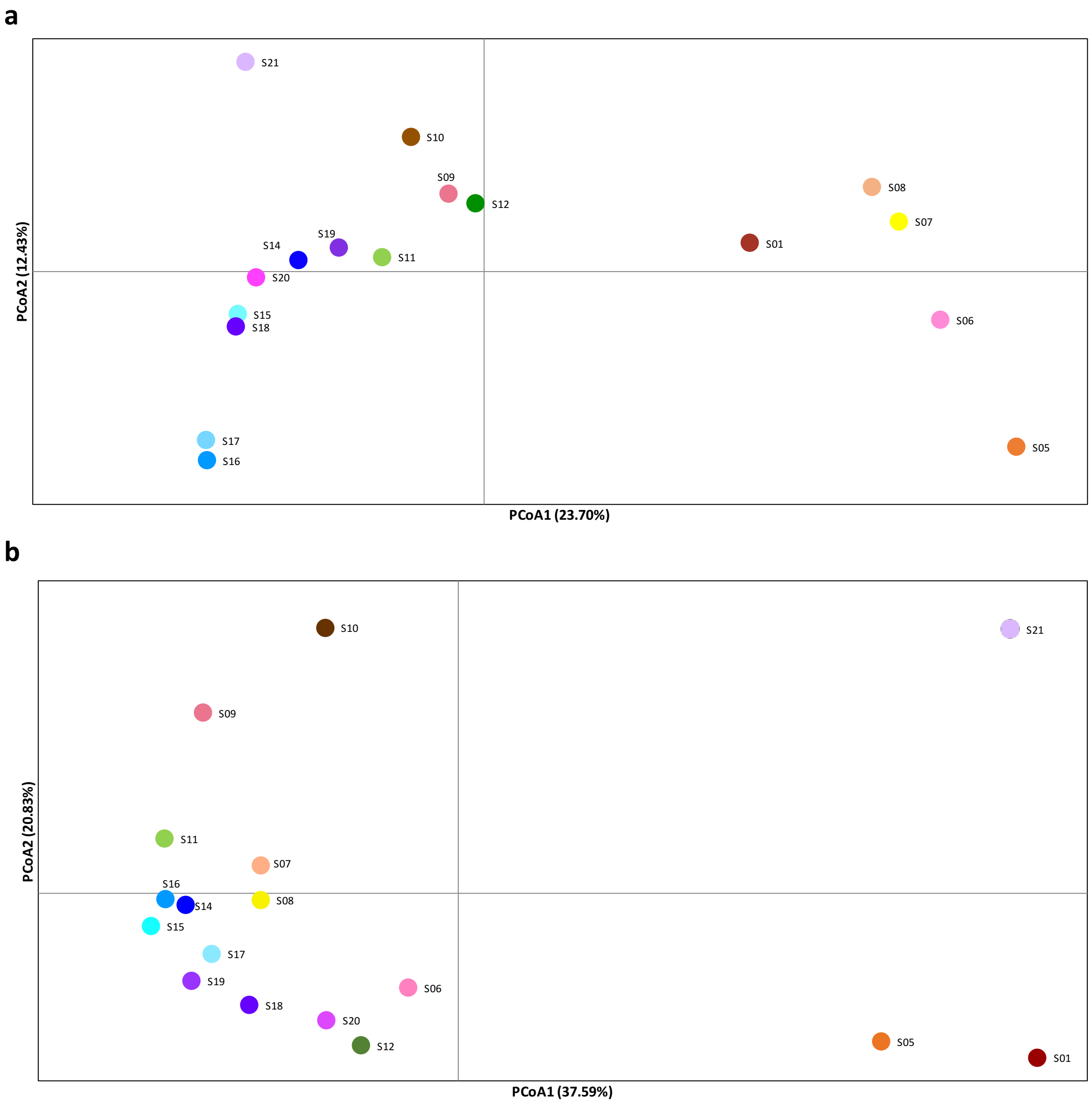

Supplementary Figure S3. Principal coordinate analysis based on pairwise F_{ST} matrices generated from non-candidate (a) and candidate climate-selected (b) loci datasets in *Carex gayana*, each composed of 90 SNPs.

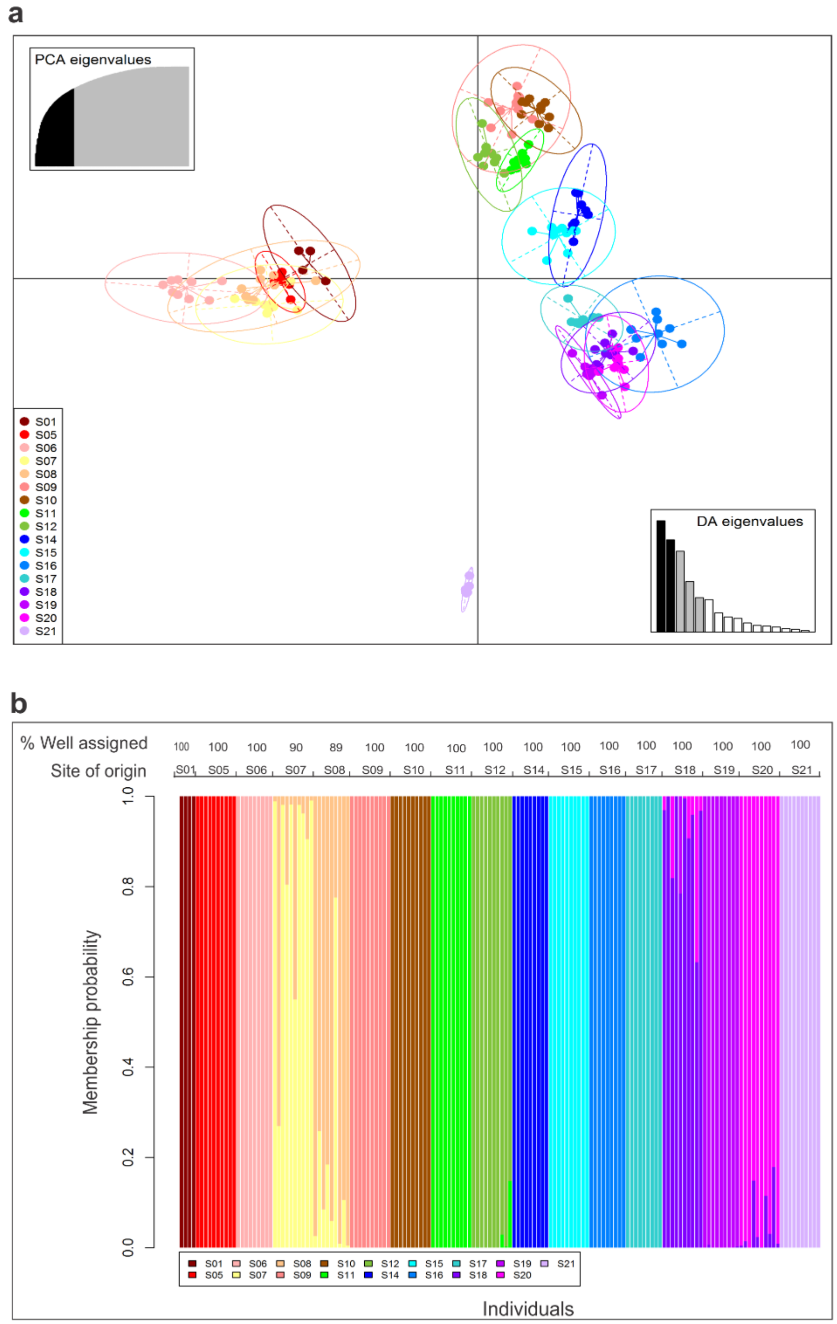

Supplementary Figure S4. Discriminant analysis of principal components (DAPC) based on the original 1421 non-candidate selected loci dataset generated for *Carex gayana*. A. Scatterplots of the discriminant analysis. Individual genotypes appear as dots. B. Bar plots of site membership probability for each *C. gayana* specimen sampled across the study area, with the percentage of specimens correctly assigned to their site of origin.

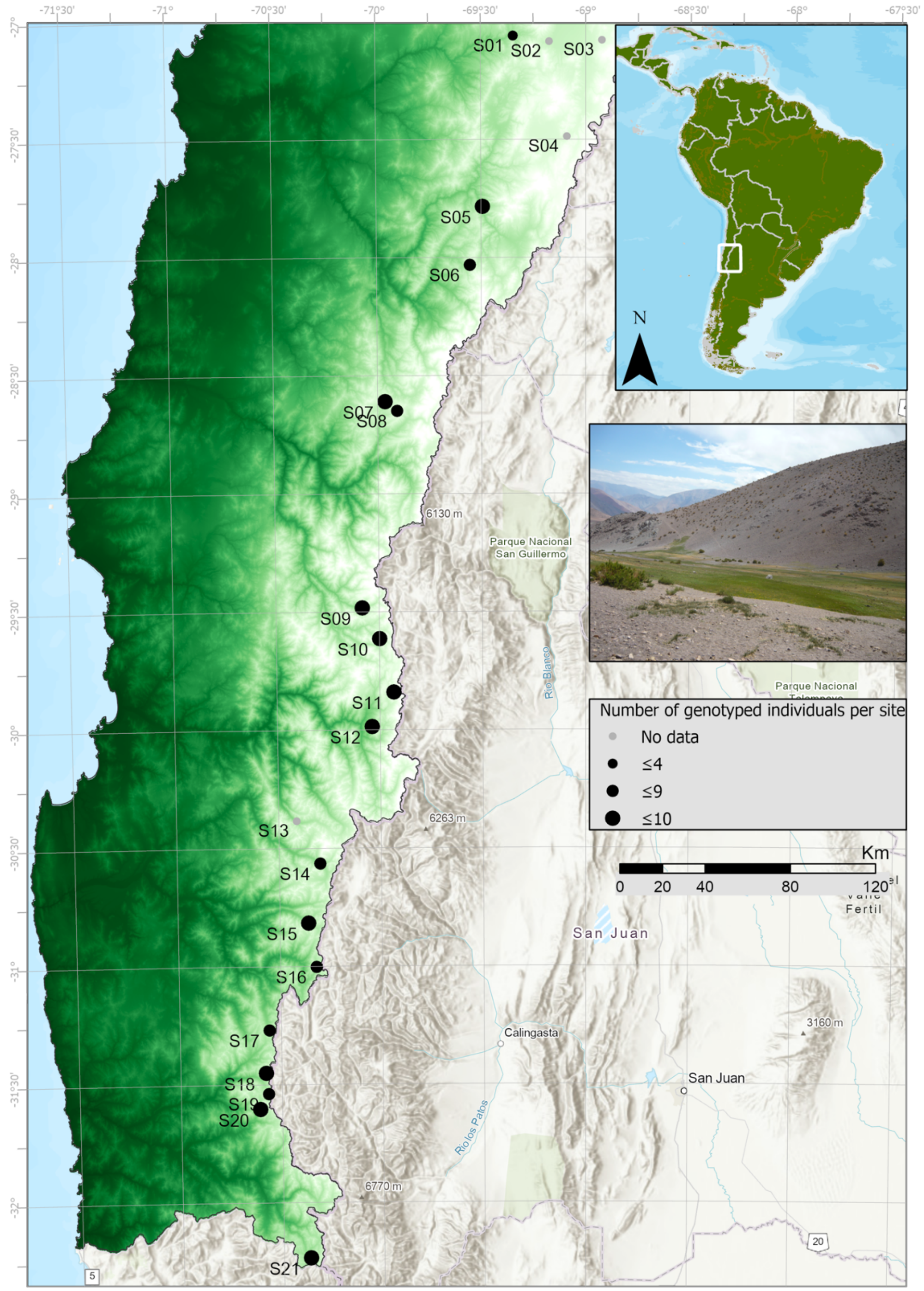

Supplementary Figure S5. Map of the study area produced using the free version of ArcGIS Pro 2.5, available for Learn ArcGIS organization at https://learn.arcgis.com and https://www.esri.com/es-cl/store/arcgis-pro; photograph at center right shows a typical high-Andean wetland in this region.

Supplementary Table S5. River basin and SNP genetic diversity estimates for the 17 *Carex gayana* sites sampled along Chile's Norte Chico. Three datasets were considered: all non-candidate selected loci (1421), 90 randomly-selected non-candidate selected loci and 90 candidate climate selected loci. *He*: unbiased expected heterozygosity.

		All non-candidate selected loci	90 non-candidate selected loci	90 candidate climate-selected loci
**Site**	**River basin**	***He***	***He***	***He***
1	Copiapó	0.071	0.090	0.094
5	Copiapó	0.083	0.057	0.132
6	Copiapó	0.088	0.101	0.089
7	Huasco	0.143	0.122	0.103
8	Huasco	0.137	0.108	0.090
9	Huasco	0.130	0.108	0.086
10	Elqui	0.121	0.088	0.070
11	Elqui	0.165	0.165	0.119
12	Elqui	0.125	0.123	0.112
14	Limarí	0.154	0.134	0.098
15	Limarí	0.155	0.166	0.090
16	Limarí	0.109	0.106	0.061
17	Limarí	0.145	0.127	0.126
18	Choapa	0.208	0.183	0.142
19	Choapa	0.232	0.216	0.103
20	Choapa	0.181	0.155	0.157
21	Choapa	0.059	0.053	0.105
**Mean value**		0.136	0.124	0.105

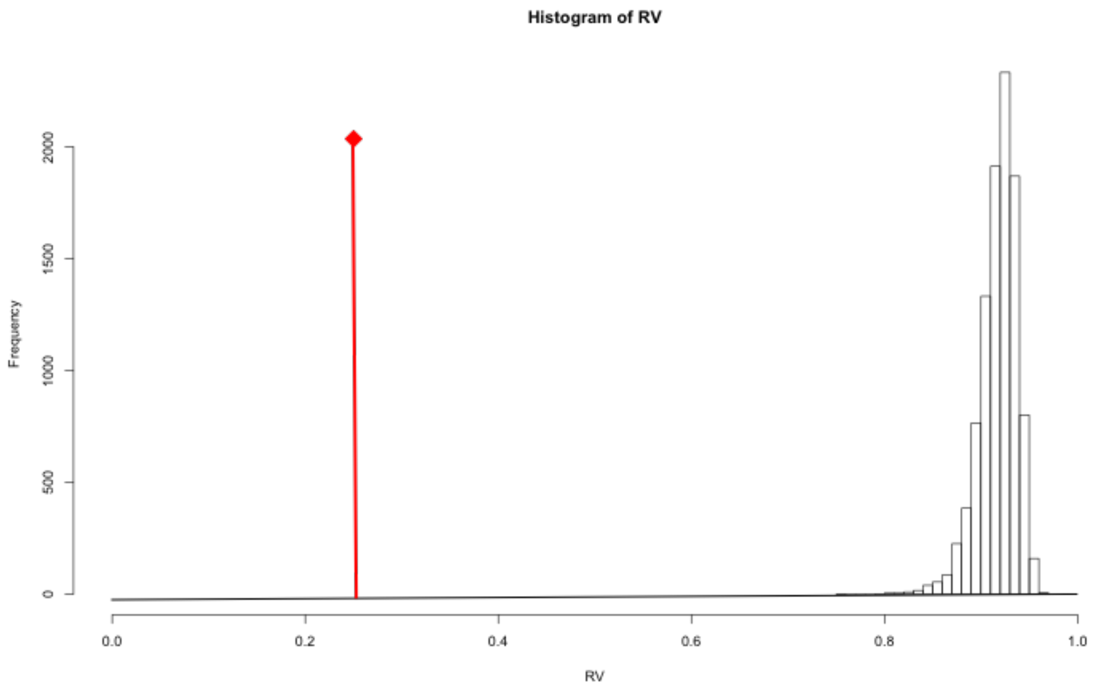

Figure S6. Bootstrap distribution of the RV coefficient calculated from 10,000 iterations and co-inertia analysis of two subsets of 90 SNPs randomly selected among the non-candidate datasets. The red line indicates the RV value calculated from the candidate and non-candidate climate-selected datasets.

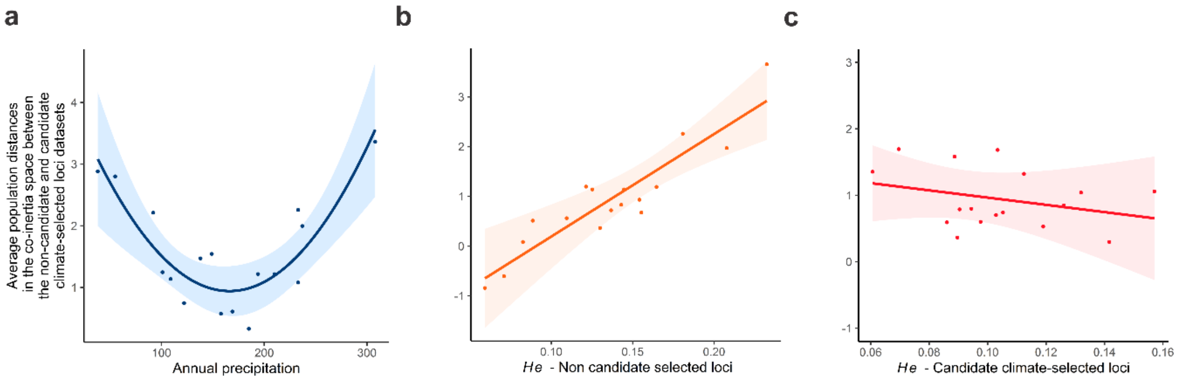

d

	$\beta \pm SD$	t	P
Annual precipitation	-0.043 ± 0.011	-4.023	<0.01
(Annual precipitation)^2	0.0001 ± 0.00003	4.297	<0.01
He – Non candidate selected loci	20.620 ± 4.356	4.733	<0.01
He – Candidate climate-selected loci	-5.453 ± 6.122	-0.891	0.391

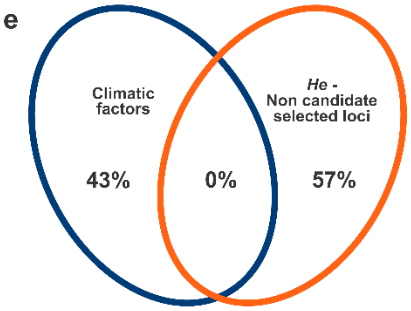

Supplementary Figure S7. Analysis of the effects of explanatory variables on mean population divergence between the candidate (i.e. 90 loci) and non-candidate climate-selected (i.e. 1421 loci) *Carex gayana* datasets, as estimated from Euclidean distances between co-inertia scores of each individual on the first seven co-inertia axes for candidate and non-candidate datasets. a,b,c,d, Conditional plots of the effects of annual precipitation, mean annual temperature, and expected heterozygosity (He) calculated from non-candidate and candidate climate-selected SNP datasets, respectively. e, Results of the regression analysis. f, Proportion of explained variance in population genetic divergence between candidate and non-candidate selected loci accounted for by significant climatic factors (annual precipitation) and genome-wide genetic diversity of the non-candidate selected loci.

References

1. Fick, S. E. & Hijmans, R. J. WorldClim 2: new 1-km spatial resolution climate surfaces for global land areas. *International Journal of Climatology* **37**, 4302-4315 (2017).
2. Vigneau, E. & Qannari, E. M. Clustering of variables around latent components. *Communications in statistics* **32**, 1131-1150 (2003).
3. Vigneau, E., Chen, M. K. & Qannari, E. M., ClustVarLV: An R package for the clustering of variables around latent variables. *R Journal* **7**, 134–148 (2015).
4. Excoffier, L. & Lischer, H. E. L. Arlequin suite ver 3.5: A new series of programs to perform population genetics analyses under Linux and Windows. *Molecular Ecology Resources* **10**, 564–567(2010).
5. Peakall, R. & Smouse, P. E. GenAlEx 6.5: genetic analysis in Excel. Population genetic software for teaching and research-an update. *Bioinformatics* **28**, 2537–2539 (2012).
6. Jombart, T., Devillard, S. & Balloux, F. Discriminant analysis of principal components: a new method for the analysis of genetically structured populations. *BMC Genetics* **11**, 94 (2010)
7. Jombart, T. & Ahmed, I. Adegenet 1.3-1: new tools for the analysis of genome-wide SNP data. *Bioinformatics* **27**, 3070–3071 (2011).
8. Troncoso, A.J., Bertin, A., Osorio, R., Arancio, G. & Gouin, N. Comparative population genetics of two dominant plant species of high Andean wetlands reveals complex evolutionary histories and conservation perspectives in Chile's Norte Chico. *Conservation Genetics* **18**, 1047–1060 (2017).
